# Relaxation Time Determines Mechanical Signal Persistence in the Periodontal Ligament Under Sustained Loading

**DOI:** 10.3390/jfb17090428

**Published:** 2026-08-25

**Authors:** Chen Zong

**Affiliations:** 1 College of Dental Medicine, Columbia University Irving Medical Center, New York, NY 10032, USA; drchenzong@outlook.com; 2 Department of Oral Health Sciences-Orthodontics, University of Leuven (KU Leuven) and Dentistry, University Hospitals Leuven, 3000 Leuven, Belgium

**Keywords:** periodontal ligament, viscoelasticity, stress relaxation, creep, finite element analysis, mechanical signal persistence, relaxation time constant, connective tissue, tissue engineering

## Abstract

Mechanical loading of connective tissues is traditionally prescribed by force magnitude. In viscoelastic tissues, a constant external force does not produce a constant internal mechanical environment: stress and strain evolve continuously after load onset, creating a time-varying tissue-level mechanical history relevant to resident cells. This distinction is rarely accounted for in clinical loading protocols or scaffold design. The rate of evolution is governed by the stress relaxation time constant (τ). How τ controls the persistence of mechanical signals under force-controlled sustained loading remains poorly quantified. Thus, we developed a three-dimensional finite element model of the Wistar rat maxillary first molar tooth–periodontal ligament (PDL)–bone complex with a PDL geometry reconstructed from micro-CT imaging by original frame-by-frame manual segmentation and compared outcomes across three τ values spanning two orders of magnitude under identical 0.5 N sustained loading. Under the same applied force, stress retention at 100 s ranged from 68% to 97%, while concurrent deformation creep showed an inverse relationship. These results demonstrate that τ strongly governs the persistence of mechanical signals under sustained force-controlled loading in this model. Supplementary simulations under oblique loading and perturbed PDL modulus confirmed that τ remains the dominant constitutive determinant of stress retention across altered loading directions and stiffness conditions.

## 1. Introduction

Connective tissues that serve load-bearing functions, including the periodontal ligament (PDL) [1] and tendon [2], share a defining mechanical property: they are viscoelastic. Under sustained loading, stress within these tissues decays progressively even when the external force remains constant, a process known as stress relaxation. Concurrent creep deformation accumulates. These responses are well documented in the PDL across multiple experimental loading protocols [3,4,5]. Their biological consequence is fundamental: cell populations within these tissues are mechanosensitive, and the stimulus they receive is not the applied force but the local stress–strain field that evolves within the tissue over time.

In clinical and tissue-engineering practice, loading protocols for connective tissue systems are characterized by applied force magnitude rather than by the resulting internal stress–strain history. For viscoelastic materials, a constant external force does not produce a constant tissue-level mechanical environment: stress and strain vary continuously throughout loading, and the rate of variation is governed by the stress relaxation time constant τ [6]. This applies to orthodontic force delivery to the PDL [7], sustained mechanical loading of tendons in rehabilitation and training [8], and mechanical conditioning protocols for tissue-engineering constructs [9]. Without a quantitative understanding of how τ controls signal persistence under sustained loading, it is not possible to predict what cells experience under a given clinical or in vitro protocol. This gap also limits rational scaffold design: the relationship between scaffold τ and the tissue-level mechanical environment under in vivo sustained loading has not been established at the structural scale.

Direct mechanical characterization of load-bearing connective tissues presents significant technical challenges. Tissue volumes are typically small, specimens cannot be isolated from their functional attachments without altering in situ mechanics, and ex vivo preparation is confounded by rapid structural changes [10]. These constraints are particularly acute for the PDL because of its small interfacial volume and its inseparable attachment to mineralized tissues. The PDL is nonetheless an excellent model system for investigating the mechanics–time relationship in viscoelastic connective tissue interfaces. Its structural context is comparatively simple: the PDL is bounded on both sides by mineralized tissue, without the mechanical contributions of overlapping muscles, tendons, or adjacent joint capsules that complicate characterization in most musculoskeletal soft tissue interfaces. The time-dependent response of the PDL under sustained orthodontic loading also represents an uncharacterized gap in the biomechanical literature. Three-dimensional finite element analysis (FEA) offers a controlled framework for addressing this question. High-resolution micro-CT of the rat dentition enables reconstruction of the tooth–bone interfacial geometry and has been validated against in vivo tooth displacement measurements [11,12]. Parametric FEA then permits systematic variation in PDL constitutive parameters while all structural and loading variables remain fixed.

Against this background, this study aims to test whether and how the stress relaxation time constant τ governs the temporal evolution of mechanical signals in a viscoelastic connective tissue under sustained force-controlled loading. Using a three-dimensional FE model of the Wistar rat maxillary first molar PDL–bone complex, we compare PDL stress, strain, and displacement outcomes across three τ values spanning distinct relaxation regimes. We further aim to determine whether a continuous τ-signal retention relationship can be derived from the simulation data and whether it offers a quantitative basis for relating relaxation kinetics to in vivo tissue-level mechanical input.

## 2. Materials and Methods

### 2.1. Animal Ethics

Wistar rat micro-CT imaging data used for model reconstruction were collected under ethics protocol P197/2019, approved by the KU Leuven Ethical Committee for Animal Experiments (valid from 1 December 2019 to 1 December 2024). No new animal procedures were conducted for this computational study.

### 2.2. Model Geometry and PDL Reconstruction

**Figure 1:**
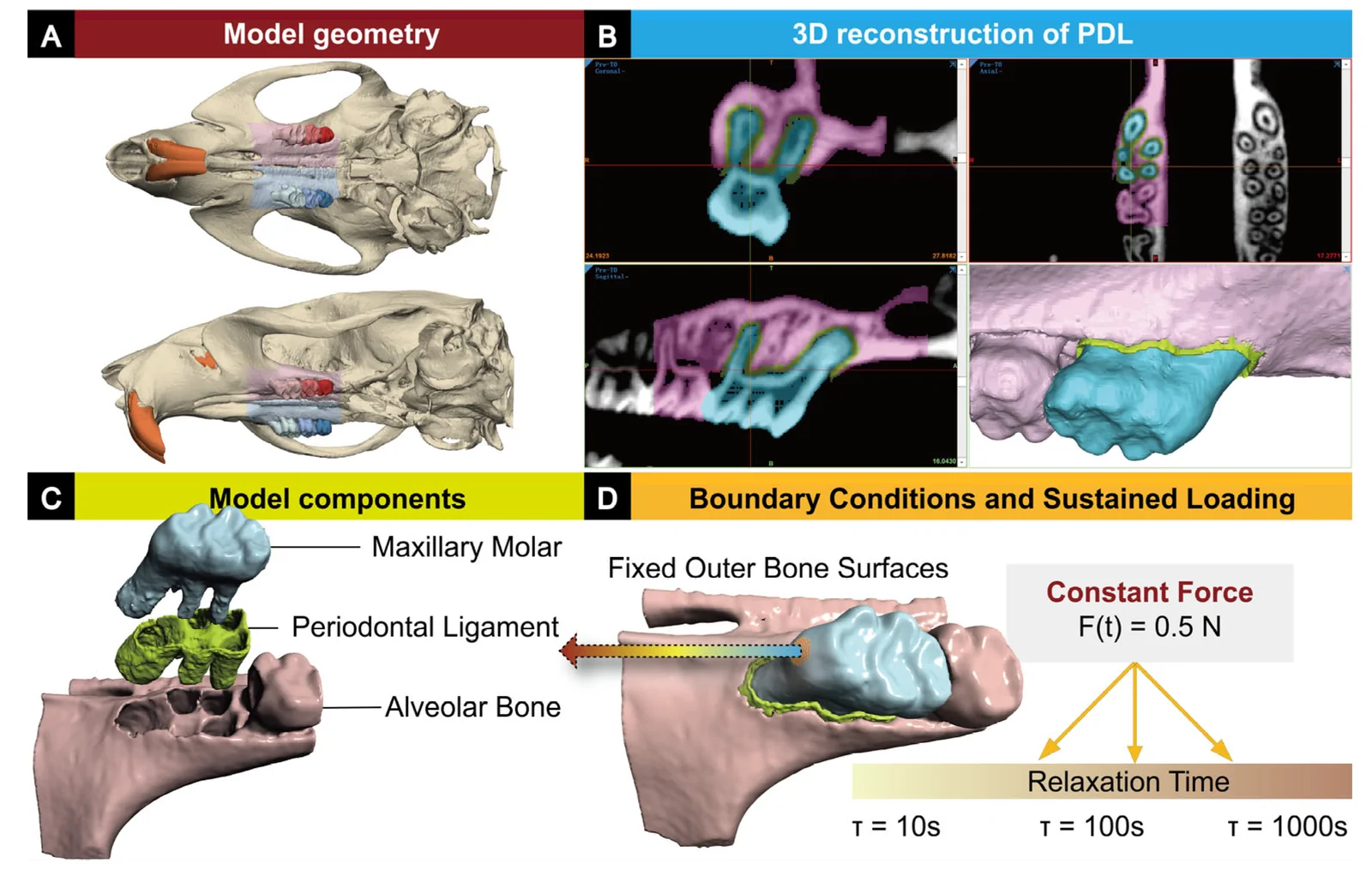
Finite element model construction. (**A**) Overview of the micro-CT-derived craniofacial geometry. (**B**) PDL segmentation workflow in Mimics 25.0: coronal, axial, and sagittal cross-sections; a rendered view of the isolated M1 root-PDL volume at lower right. (**C**) Exploded view of the three model components: maxillary molar, PDL, and alveolar bone. (**D**) Assembled model with boundary conditions: outer bone surfaces are fully fixed; a distributed surface load with resultant intrusive force F(t) = 0.5 N is applied to the occlusal crown surface; three parametric τ cases (10, 100, 1000 s) are simulated under identical loading.

The three-dimensional geometry of the Wistar rat maxillary first molar (M1) tooth and surrounding alveolar bone was reconstructed from micro-CT data (55 μm isotropic voxel resolution) using Mimics 25.0 (Materialise NV, Leuven, Belgium). Micro-CT scanning, image acquisition, and volume reconstruction protocols were as described previously [11,12]. Segmentation of tooth and alveolar bone was performed by intensity thresholding with manual slice-by-slice correction, following procedures previously developed for rat dental micro-CT datasets [13,14]. The model geometry, PDL domain, mesh, and boundary conditions are illustrated in Figure 1.

For the PDL domain, an original manual segmentation procedure was developed because established segmentation protocols were not directly applicable to resolving the PDL interface at 55 μm voxel resolution. The root surface segmentation was first expanded by one voxel to generate an initial candidate PDL layer. This layer was then refined in Mimics 25.0 frame by frame across all three orthogonal planes, removing each voxel in contact with the tooth or bone until the PDL volume was free of spatial overlap at every cross-section. Validation was performed using Boolean intersection analysis: the PDL volume shared zero voxels with the tooth segmentation and with the bone segmentation, confirming mutually exclusive voxel assignments among tooth, PDL, and bone labels. The union of tooth, PDL, and bone volumes matched the predefined interfacial region of interest without remainder, confirming spatial completeness. Geometry was exported to 3-matic 17.0 (Materialise NV, Leuven, Belgium) for surface remeshing, smoothing, and finite element pre-processing. The tetrahedral mesh (459,708 nodes; 2,547,485 four-node elements) was generated in 3-matic 17.0 and verified using Jacobian and aspect-ratio quality inspections (no failed elements reported).

### 2.3. Material Constitutive Models

Alveolar bone and tooth structure were modeled as linearly isotropic elastic continua, with material parameters for bone and tooth drawn from reported literature values [15,16] (Table 1). The PDL was modeled as a linear viscoelastic solid using a single-term Prony series shear relaxation function, following the formulation established by Fill et al. [10] and applied in previous PDL viscoelastic finite element analyses [17]:

*G*(*t*) = *G*_0_ [*g*_0_ + *g*_1_
*exp*(−*t*/*τ*_1_)]

where *G*_0_ is the instantaneous shear modulus derived from E_0_ = 0.20 MPa and ν = 0.45, *g*_0_ = *g*_1_ = 0.50, and *τ*_1_ is the relaxation time constant. Because a single-term Prony series was used, *τ*_1_ is hereafter referred to as *τ*. The coefficients *g*_0_ = *g*_1_ = 0.50 were chosen to provide a 50% long-term relaxation amplitude for controlled parametric comparison, within the range reported in the literature [4,5]. All material parameters are listed in Table 1.

### 2.4. Boundary Conditions and Loading

Zero-displacement constraints were imposed on all outer alveolar bone surfaces. Tooth–PDL and PDL–bone interfaces were modeled using tied-elastic contact [18]. A sustained distributed surface load with a resultant intrusive force of 0.5 N was applied to the crown occlusal surface at t = 0 and maintained constant for 100 s. This force magnitude matches that used in rat orthodontic loading experiments from the same model system [14].

### 2.5. Simulation Protocol and Output Metrics

Simulations were prepared and post-processed using FEBio Studio 3.1.0 (University of Utah, Salt Lake City, UT, USA) and solved using the FEBio 4.12.0 finite element solver (University of Utah, Salt Lake City, UT, USA), an open-source nonlinear FE solver developed and validated for computational biomechanics [19]. A fully implicit transient viscoelastic analysis was used with a 100 s duration, 10 equal steps of 10 s each, a displacement convergence norm of 0.001, and an energy convergence norm of 0.01. Three parametric cases were defined, differing only in τ. Values of τ = 10, 100, and 1000 s were selected to span two orders of magnitude within the range reported for PDL and analogous connective tissue relaxation [3,10]. Published Prony-series fits to PDL creep and relaxation data report τ values ranging from approximately 1 s to over 1000 s, depending on species, loading mode, strain level, and the number of Prony terms employed [1,4,5,10,20]. The three selected values represent rapid-relaxation, intermediate, and quasi-elastic regimes relative to the 100 s observation window and are not intended to represent discrete biological phenotypes.

At each output state, three scalar metrics were extracted from the PDL domain: maximum von Mises (VM) stress σ_VM, maximum principal strain ε, and maximum nodal displacement magnitude. Time histories of σ_VM and ε were normalized to their respective values at t = 10 s (the first recorded output state). Stress retention is defined as R(100 s) = σ_VM(100 s)/σ_VM(10 s). PDL displacement creep is the percentage change in maximum nodal displacement from t = 10 to 100 s. Strain creep was calculated analogously as the percentage change in maximum principal strain between t = 10 and 100 s. The continuous τ-retention relationship was derived by fitting a log-quadratic function R(τ) = a(log_10_τ)^2^ + b(log_10_τ) + c to the three simulation points via an exact algebraic solution of the resulting 3 × 3 linear system (coefficients: a = 0.04250, b = −0.02550, c = 0.66300). The function passes exactly through all three simulation points and is an interpolation, not a regression. All simulations are deterministic parametric analyses with no experimental replication; no inferential statistical testing was performed. Quantitative results are summarized in Table 2.

### 2.6. Robustness and Sensitivity Analyses

To assess the robustness of the τ–retention relationship, two supplementary analyses were performed using the same mesh, boundary conditions, and simulation protocol.

*Loading direction robustness*: Three additional simulations were conducted with a 45° oblique force (resultant 0.5 N, applied as Fx = 0.354 N, Fy = −0.354 N on the occlusal surface), combining intrusive and buccolingual components. All three τ values (10, 100, 1000 s) were tested under this oblique loading condition.

*PDL modulus perturbation*: Two simulations varied the instantaneous PDL Young’s modulus E_0_ by ±25% from the baseline value (E_0_ = 0.15 MPa and E_0_ = 0.25 MPa), with *τ*_1_ fixed at 100 s and all other parameters unchanged. This range was selected to represent a plausible degree of inter-individual and inter-study variability in reported PDL elastic properties [10,15,16]. The metric R(100 s) was compared across all conditions to determine whether the effect of τ variation exceeded the effect of modulus variation within these ranges.

## 3. Results

### 3.1. Simulation Convergence

All three cases terminated normally using the FEBio 4.12.0 solver (1,374,792 active degrees of freedom; 2–3 Newton–Raphson iterations per step). Convergence behavior was identical across all τ values, confirming that output differences reflect constitutive variation alone.

### 3.2. Stress Relaxation Rate and Extent Are Governed by τ

**Figure 2:**
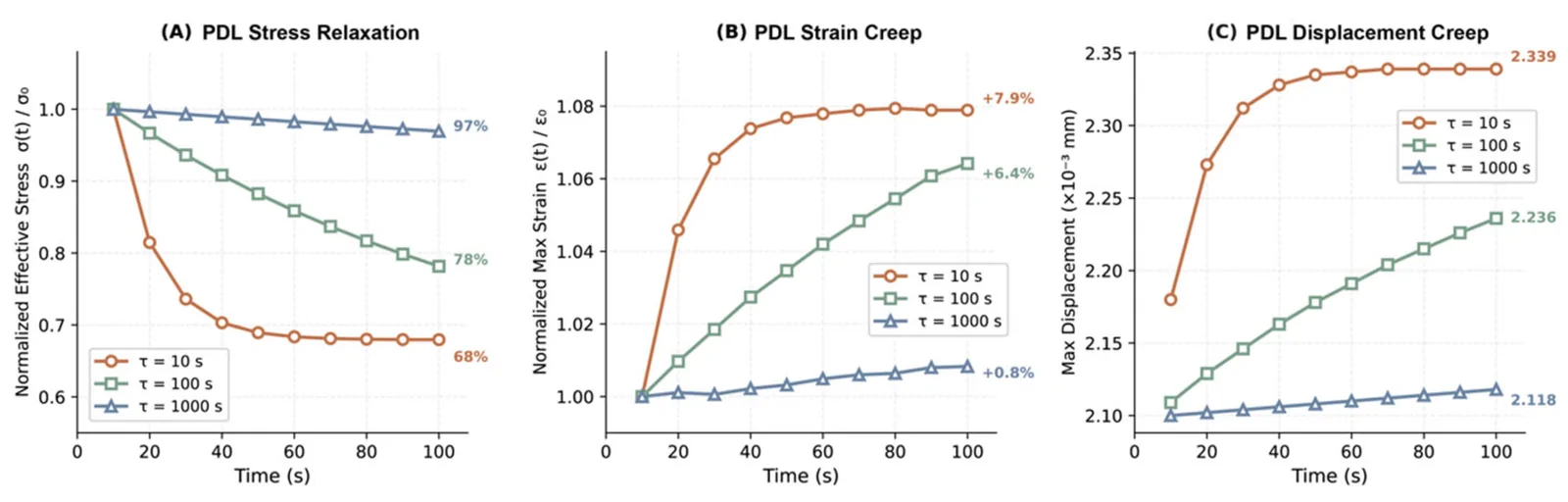
Time-dependent PDL mechanical response under sustained 0.5 N intrusive loading for three relaxation time constants: τ = 10 s (orange circles), τ = 100 s (green squares), τ = 1000 s (blue triangles). Lines connect consecutive FEBio output states (10 equal time steps). (**A**) Normalized von Mises stress σ(t)/σ_0_ (normalized to the value at t = 10 s); stress retention R(100 s) annotated. (**B**) Normalized maximum principal strain ε(t)/ε_0_; cumulative creep percentage annotated. (**C**) Absolute maximum nodal displacement magnitude in the PDL (×10^−3^ mm); final values at t = 100 s annotated.

Under a constant 0.5 N load, PDL von Mises stress decreased continuously throughout the 100 s window in all three groups, confirming that a nominally constant external force does not produce a constant internal stress state in a viscoelastic tissue. The rate and extent of decay were strongly τ-dependent (Figure 2A). The fastest-relaxing PDL reached a near-plateau early in the loading window; the slowest-relaxing PDL showed a near-linear decline throughout. Stress retention at 100 s ranged from 68% for the fastest group to 97% for the slowest.

Peak stress at t = 10 s was itself τ-dependent: lowest for the fastest-relaxing PDL because relaxation is largely complete within one time constant and highest for the slowest. Quantitative values are in Table 2. Static elastic FEA, which reports only the instantaneous response, may therefore overestimate the sustained tissue-level stress relevant to cellular mechanosensing in fast-relaxing tissues.

### 3.3. Faster Relaxation Accumulates Greater Creep Deformation

Concurrent with stress decay, PDL deformation increased progressively over time. Faster-relaxing PDL accumulated substantially greater displacement and principal strain creep than slower-relaxing PDL over the 100 s window (Figure 2B,C; Table 2). Creep magnitude differed by nearly an order of magnitude between the extreme groups. This inverse relationship between stress retention and creep accumulation constitutes a stress-deformation decoupling: under identical applied forces, faster relaxation simultaneously lowers sustained stress and amplifies geometric change; slower relaxation preserves elevated stress with minimal creep.

### 3.4. Spatial Distribution Is Anatomy-Governed; Magnitudes Are τ-Governed

**Figure 3:**
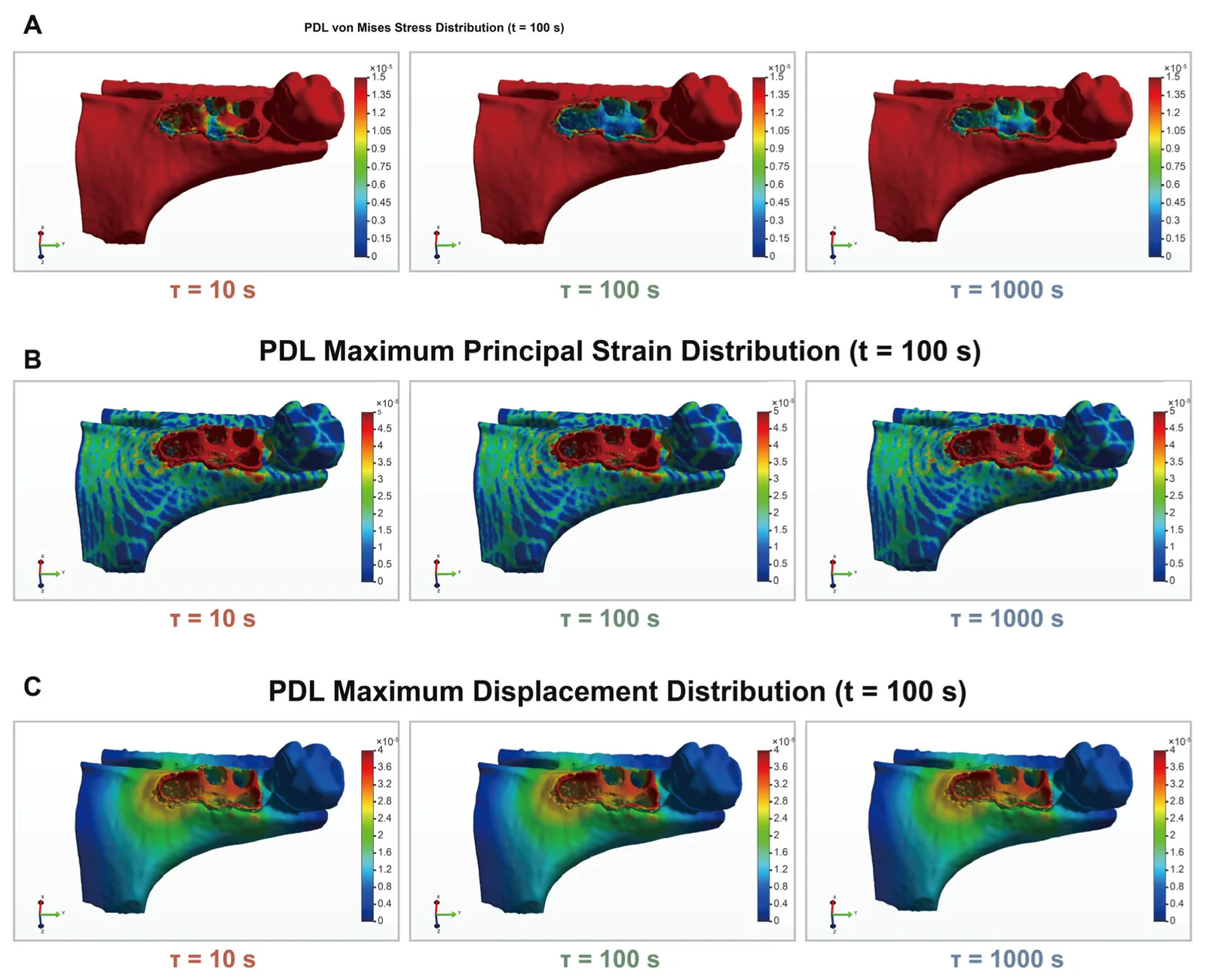
Spatial distribution of mechanical fields across the model at t = 100 s for three τ values. (**A**) Von Mises effective stress (MPa). (**B**) Maximum principal strain. (**C**) Nodal displacement magnitude (mm). Each row shares a color scale across the three τ columns. Spatial concentration patterns are anatomy-governed and qualitatively consistent across τ groups; τ-dependent magnitude differences are quantified in Table 2.

Spatial distributions at t = 100 s are shown in Figure 3. Patterns of von Mises stress, principal strain, and nodal displacement were qualitatively identical across all three τ groups. Concentrations localized to the cervical PDL adjacent to the alveolar crest, with displacement decreasing apically. This distribution is determined by the shared anatomical geometry and is invariant across τ. The τ-dependent differences manifest as magnitude shifts at these fixed concentration zones. All time-dependent mechanical changes over the 100 s window were accommodated primarily within the compliant PDL interface, consistent with the high stiffness contrast between the tooth and the PDL.

### 3.5. Continuous τ-Retention Relationship

**Figure 4:**
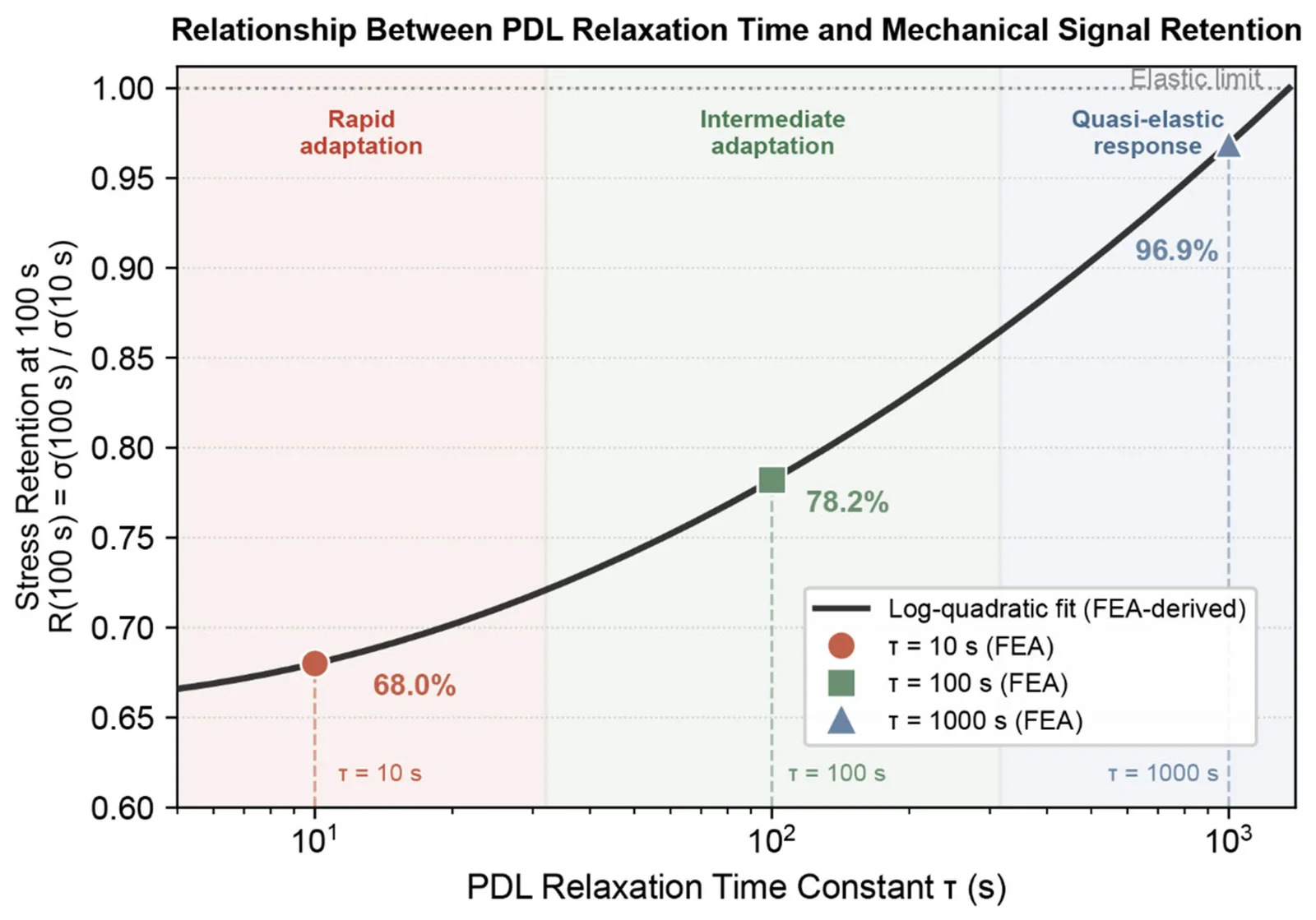
Relationship between PDL relaxation time constant τ and mechanical signal retention R(100 s) = σ(100 s)/σ(10 s). Symbols: FEA simulation results at τ = 10 s (orange circle, 68.0%), 100 s (green square, 78.2%), and 1000 s (blue triangle, 96.9%). Solid curve: log-quadratic interpolation through the three simulation points (coefficients a = 0.04250, b = −0.02550, c = 0.66300). Dotted horizontal line: elastic limit (R = 1.0, τ → ∞). Shaded regions indicate three illustrative operational regimes: rapid relaxation (τ < 30 s), intermediate relaxation (30–300 s), and quasi-elastic response (τ > 300 s); regime boundaries are illustrative, not statistically derived.

To place the three parametric cases within a continuous framework, R(100 s) is plotted as a function of τ in Figure 4. The three simulation points were interpolated by a log-quadratic function (Section 2.5). Because simulations used force-controlled loading rather than idealized strain-controlled relaxation, the τ-retention relationship is derived from FE results rather than from the analytical Prony function. For visual interpretation, three illustrative operational regimes are indicated: rapid adaptation (τ < 30 s), intermediate adaptation (30–300 s), and quasi-elastic response (τ > 300 s). The choice of these boundaries is illustrative and is not derived from statistical analysis.

### 3.6. Stress Retention Under Oblique Loading

**Figure 5:**
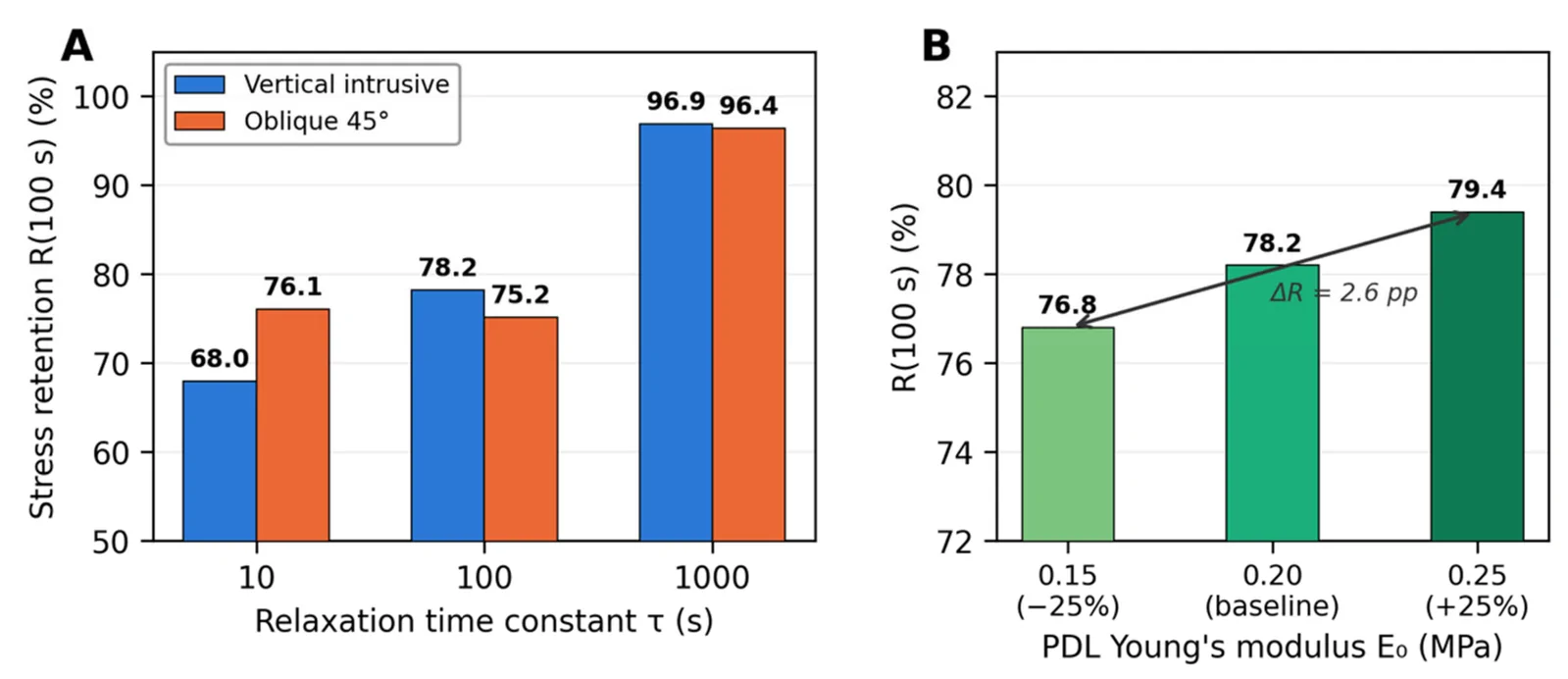
Robustness and sensitivity analysis of the τ–retention relationship. (**A**) Stress retention R(100 s) under vertical intrusive loading (blue bars) and 45° oblique loading (orange bars) for three relaxation time constants (τ = 10, 100, 1000 s). Despite changes in absolute stress magnitude and spatial distribution under oblique loading, the substantially higher retention at τ = 1000 s is preserved. (**B**) Effect of PDL instantaneous Young’s modulus E_0_ perturbation on R(100 s) at fixed τ = 100 s. Varying E_0_ by ±25% from the baseline (0.20 MPa) produced a total variation of 2.6 percentage points in R(100 s) (76.8% to 79.4%), compared to 29 percentage points across the two-order-of-magnitude τ range at baseline E_0_ (68.0% to 96.9%), indicating that τ produced a larger change in stress retention within the tested parameter intervals.

Under the 45° oblique force, peak PDL von Mises stress at t = 10 s was lower than under vertical intrusive loading for all three τ groups (Table 3), reflecting the redistribution of stress under a combined intrusive–buccolingual load state. Despite the change in stress magnitude and spatial distribution, the pattern of substantially greater stress retention at τ = 1000 s was preserved, whereas the τ = 10 and τ = 100 s cases showed similar retention under oblique loading (Figure 5A). Longer relaxation times continued to sustain higher proportions of the initial stress, indicating that the influence of τ on stress retention extends beyond the purely intrusive loading condition within this model.

### 3.7. PDL Modulus Perturbation

Varying E_0_ by ± 25% at fixed τ = 100 s produced R(100 s) values of 76.8% (E_0_ = 0.15 MPa) and 79.4% (E_0_ = 0.25 MPa), compared to 78.2% at the baseline E_0_ = 0.20 MPa (Figure 5B). The maximum variation in R(100 s) attributable to a 50% range in E_0_ was 2.6 percentage points. By contrast, varying τ from 10 to 1000 s at baseline E_0_ produced a 29-percentage-point change in R(100 s) (68.0% to 96.9%). Thus, within the selected parameter intervals, variation in τ produced a larger change in stress retention than the tested E_0_ perturbation, although this comparison does not represent a normalized sensitivity index.

## 4. Discussion

### 4.1. PDL Reconstruction Fidelity and the Value of Anatomically Faithful Interface Modeling

Prior finite element studies of the tooth–bone complex have commonly represented the PDL as a layer of uniform prescribed thickness, generated by a constant offset from the root surface [21]. Natali et al. formulated a nonlinear constitutive law for the PDL within an analytically constructed tooth model [22]. Pietrzak et al. defined the PDL geometry by prescribed interfacial boundary coordinates within a simplified molar geometry [23]. Porohyperelastic formulations have been implemented within comparably idealized PDL domains [24]. In each case, the spatial stress distribution within the PDL reflects the assumed geometric layer rather than the actual anatomical interfacial volume.

In the present study, the PDL domain was reconstructed from the micro-CT grayscale volume by original frame-by-frame manual segmentation validated by zero-voxel-overlap Boolean intersection analysis. This approach provides a geometry based on the reconstructed micro-CT interfacial anatomy, offering an anatomically informed alternative to the assumed uniform-thickness PDL layers. The spatial distribution outcomes reported here are determined by the reconstructed tissue geometry rather than by modeling assumptions.

An important consequence of the anatomically reconstructed geometry is the deformation partitioning result: all time-dependent mechanical changes were accommodated primarily at the PDL interface, consistent with the high stiffness contrast between the tooth and the PDL. This partitioning reflects the extreme stiffness contrast between the tooth and the PDL [25] and is consistent with experimental evidence that early orthodontic tooth displacement is dominated by PDL interface mechanics rather than alveolar bone bending [11]. It confirms that the PDL domain, as reconstructed, is the principal locus of mechanical signal generation under sustained orthodontic loading.

Our predicted displacement and stress magnitudes are consistent in order of magnitude with values reported in prior tooth–PDL–bone FEA studies under comparable loading conditions [16,17,21], although direct comparison is precluded by differences in species, geometry, and constitutive models.

### 4.2. A Constant Applied Force Does Not Produce a Constant Mechanosensory Environment

The fundamental finding of this study extends beyond orthodontics. In principle, in a load-bearing viscoelastic connective tissue, a constant external force generates a continuously evolving internal mechanical state. The effective stimulus available to tissue-resident cells is not the applied force but the stress–strain history that accumulates within the tissue over time. This distinction is largely absent from clinical loading protocols for connective tissue systems.

Standard orthodontic practice characterizes force delivery by magnitude and direction. The implicit premise is that a specified force translates to a specified cellular stimulus. This premise does not hold for viscoelastic tissues. As shown in Figure 4, stress retention at 100 s ranged from 68% to 97% across the τ range examined under identical applied forces. Cells in a fast-relaxing PDL would be expected to experience a brief, high-amplitude mechanical pulse followed by a progressively declining stress environment. The tissue-level mechanical stimulus relevant to mechanosensing would then decay to a fraction of its initial value within one time constant. Cells in a slow-relaxing PDL sustain near-elastic mechanical conditions throughout the same loading interval [26]. These are mechanobiologically distinct situations under a clinically identical force prescription.

The present simulations show that τ strongly governed the temporal evolution of mechanical signals in a viscoelastic connective tissue under sustained force-controlled loading. PDL cell mechanotransduction involves responses to local strain amplitude and duration rather than to applied force [27]. Sustained mechanical loading produces distinct biological outcomes compared to brief-peak stimulation in periodontal tissue [28]. Characterizing a loading protocol by force magnitude alone provides an incomplete description of the cellular stimulus.

### 4.3. PDL-Dominated Deformation, Stress-Deformation Decoupling, and Structural Consequences

The supplementary oblique-loading simulations further examined whether the influence of τ observed under intrusive loading persists under a different stress state. Under a 45° oblique force combining intrusive and buccolingual components, τ = 1000 s remained clearly distinguishable from shorter relaxation times, whereas τ = 10 s and τ = 100 s showed comparable retention values. This observation indicates that relaxation time remains an important factor controlling stress persistence under altered loading conditions, although the precise ordering among shorter relaxation times may depend on the applied loading configuration.

The modulus perturbation analysis provides further evidence that τ is the dominant constitutive determinant of stress retention in this model. A ± 25% variation in E_0_—representative of inter-study variability in reported PDL elastic properties—produced only a 2.6-percentage-point change in R(100 s) (76.8% to 79.4%), whereas the two-order-of-magnitude variation in τ produced a 29-percentage-point change (68.0% to 96.9%). Thus, within the selected parameter intervals, variation in τ produced a larger change in stress retention than the tested E_0_ perturbation, although this comparison does not represent a normalized sensitivity analysis. A comprehensive global sensitivity analysis encompassing all geometric, material, and boundary-condition parameters would be valuable but constitutes an independent methodological study; the present targeted analysis demonstrates that within the tested constitutive framework, τ exerts a substantially larger effect on mechanical signal persistence than the instantaneous elastic modulus.

A further consequence of viscoelastic behavior under force-controlled loading is the inverse coupling between stress retention and deformation accumulation. Faster relaxation simultaneously reduced retained stress and amplified displacement creep. These two variables moved in opposite directions under identical applied forces. This stress-deformation decoupling means that neither peak stress nor gross tooth displacement alone can serve as a proxy for PDL mechanical input under sustained loading. Both must be resolved, and both depend on τ.

Macroscopic tooth position measurements made during or shortly after force application do not capture the PDL interface deformation that is mechanobiologically relevant. The tissue-level mechanical state is more directly assessed by resolving the PDL interface, as done in the present study.

Spatially, stress and strain concentrated consistently at the cervical PDL adjacent to the alveolar bone crest across all three τ groups. This pattern is anatomy-governed and τ-invariant in its spatial character. The mechanical exposure of this clinically vulnerable cervical PDL region is τ-sensitive in magnitude; however, under identical applied force, the sustained stress retained in the cervical PDL at 100 s differed by nearly 30 percentage points between the extreme τ groups.

### 4.4. Broader Implications for Relaxation Kinetics as a Design Parameter

The τ-retention framework quantified here may inform the design of viscoelastic biomaterial scaffolds for PDL regeneration. Extracellular matrix viscoelasticity regulates cell differentiation, matrix organization, and tissue morphogenesis [29]. The τ-retention curve in Figure 4 provides a tissue-scale rationale: a scaffold with τ in the rapid-relaxation regime delivers a brief, decaying stress to encapsulated cells under sustained loading, while one in the quasi-elastic regime sustains near-elastic stress throughout. This distinction cannot be inferred from static stiffness measurements alone. Scaffolds with target relaxation kinetics rather than target static stiffness are increasingly reported for PDL repair [30]. Cell-level mechanical dosage under sustained loading depends on the scaffold’s relaxation timescale in the PDL-relevant biomaterial system [31,32].

The same reasoning applies more broadly. In musculoskeletal rehabilitation, the relaxation kinetics of healing tendon or ligament repair tissue may influence how much of the applied tensile stimulus persists as cellular mechanostimulation during a sustained loading session. In distraction osteogenesis, the regenerating callus’s relaxation character may influence osteoprogenitor mechanobiology. In each case, τ links applied force to the tissue-level mechanical history relevant to cellular mechanosensing.

### 4.5. Limitations

The PDL was modeled as a homogeneous linear viscoelastic continuum with a single Prony relaxation term. Native PDL is fiber-reinforced, anisotropic, and nonlinearly strain-stiffening, with an interstitial fluid phase contributing to the time-dependent response [20,33]. Prony parameters were selected to span distinct relaxation regimes rather than from direct measurement on Wistar rat PDL. The 55 μm voxel resolution limits representation of sub-voxel PDL thickness variation. Experimental validation of simulated stress and strain values against in vivo measurements remains an important direction for future work. The 100 s window captures only the early viscoelastic transient and does not model cyclic loading, long-term biological adaptation, or bone remodeling.

## 5. Conclusions

Three conclusions emerge from this study. First, a nominally constant applied force does not produce a constant mechanical environment in a viscoelastic connective tissue: stress decays continuously after load onset, and the time-integrated stress–strain history within the tissue is governed by τ rather than by the applied force alone. Second, τ is the dominant material determinant of mechanical signal persistence within the tested constitutive framework: across two orders of magnitude in τ, stress retention at 100 s ranged from 68% to 97% under identical loading, while deformation creep diverged inversely. Supplementary analyses under oblique loading and with perturbed PDL modulus further demonstrated that the influence of τ on stress retention remained evident under altered loading direction and instantaneous stiffness within the tested ranges. Third, the quantitative τ–retention relationship derived from these simulations provides a tissue-scale framework for relating relaxation kinetics to sustained mechanical input. This offers a quantitative basis for specifying the target viscoelastic properties of next-generation PDL-repair scaffolds and, more broadly, for characterizing mechanical dosage in force-controlled loading of any viscoelastic connective tissue.

## Figures and Tables

**Table 1:** Material constitutive parameters adopted in the FE model.

Tissue	E (MPa)	ν	ρ (Tonne/mm^3^)	Constitutive Model
Alveolar bone	13,700	0.30	1.8 × 10^−9^	Linear isotropic elastic
Tooth structure	18,000	0.30	2.0 × 10^−9^	Linear isotropic elastic
PDL	0.20 (E_0_)	0.45	1.0 × 10^−9^	Prony series viscoelastic; *g*_0_ = *g*_1_ = 0.50; *τ*_1_ = 10, 100, or 1000 s

**Table 2:** PDL mechanical output metrics. R(100 s): stress retention at 100 s; creep calculated from t = 10 to 100 s.

Category	Parameter	τ = 10 s	τ = 100 s	τ = 1000 s
Stress	Peak σ_VM at t = 10 s (MPa)	0.01608	0.01833	0.01861
	σ_VM at t = 100 s (MPa)	0.01093	0.01433	0.01804
	Stress retention R(100 s)	68.0%	78.2%	96.9%
Deformation	PDL strain creep, Δε (t = 10 → 100 s)	+7.9%	+6.4%	+0.8%
	PDL displacement creep, Δu (t = 10 → 100 s)	+7.3%	+6.0%	+0.9%

**Table 3:** Stress retention R(100 s) under vertical and oblique loading and under PDL modulus perturbation.

Condition	τ (s)	E_0_ (MPa)	σ_VM(10 s) MPa	σ_VM(100 s) MPa	R(100 s)
Vertical	10	0.20	0.01608	0.01093	68.0%
Vertical	100	0.20	0.01833	0.01433	78.2%
Vertical	1000	0.20	0.01861	0.01804	96.9%
Oblique 45°	10	0.20	0.01012	0.00770	76.1%
Oblique 45°	100	0.20	0.00844	0.00635	75.2%
Oblique 45°	1000	0.20	0.00859	0.00829	96.5%
Vertical	100	0.15	0.01481	0.01137	76.8%
Vertical	100	0.25	0.02138	0.01698	79.4%

## Data Availability

The original contributions presented in the study are included in the article, further inquiries can be directed to the corresponding author.
